# Hydralazine-Induced Vasculitis: Case Insights and Literature Review for Informed Clinical Recognition and Management

**DOI:** 10.7759/cureus.50338

**Published:** 2023-12-11

**Authors:** Divya Shah

**Affiliations:** 1 Internal Medicine, University of Arizona College of Medicine - Phoenix, Phoenix, USA

**Keywords:** anca-associated vasculitis (aav), anca-associated vasculitis, small vessel vasculitis, lekocytoclastic vasculitis, hydralazine-induced pancytopenia, hydralazine-induced vasculitis

## Abstract

Hydralazine, a commonly prescribed medication for hypertension, has been associated, albeit rarely, with the development of vasculitis. The case presentation involves a 51-year-old female with diabetes and hypertension who presented with a distinctive rash, pancytopenia, and positive findings for various antibodies. A collaborative approach involving rheumatology, hematology, and dermatology was crucial in diagnosing leukocytoclastic vasculitis attributed to hydralazine. Prompt discontinuation of hydralazine and the initiation of a tailored treatment plan led to favorable outcomes. The study sheds light on the clinical manifestations, diagnostic challenges, and management strategies associated with this rare side effect, providing valuable insights for healthcare providers. Increased awareness of hydralazine-induced vasculitis is crucial for early recognition and proper management, ultimately contributing to improved patient outcomes in hypertension management.

## Introduction

Hydralazine is a medication commonly prescribed to treat high blood pressure. As a vasodilator, it relaxes and widens blood vessels, improving blood flow and reducing blood pressure [1]. Despite its general tolerance, like all medications, hydralazine may yield side effects such as headaches, nausea, and a rapid heartbeat. Notably, albeit rarely, there have been associations between hydralazine usage and the onset of vasculitis [2]. Vasculitis, a complex condition marked by blood vessel inflammation, poses diagnostic challenges due to its symptoms overlapping with autoimmune disorders like systemic lupus erythematosus [2]. Symptoms encompass fatigue, fever, weight loss, and diverse skin manifestations. Distinguishing vasculitis from other ailments requires a meticulous examination and comprehensive exploration of the patient’s medical history, specifically focusing on medication records, given the potential of certain drugs to induce vasculitis. Diagnosis involves clinical assessments, inflammatory marker blood tests, imaging studies, and, at times, tissue biopsies. Given the diverse impact of vasculitis on various organ systems, accurate diagnosis and effective management often necessitate collaboration among rheumatologists, dermatologists, and other specialists. Timely identification and intervention are crucial to preventing complications and improving outcomes for individuals with vasculitis.

## Case presentation

**Figure 1:**
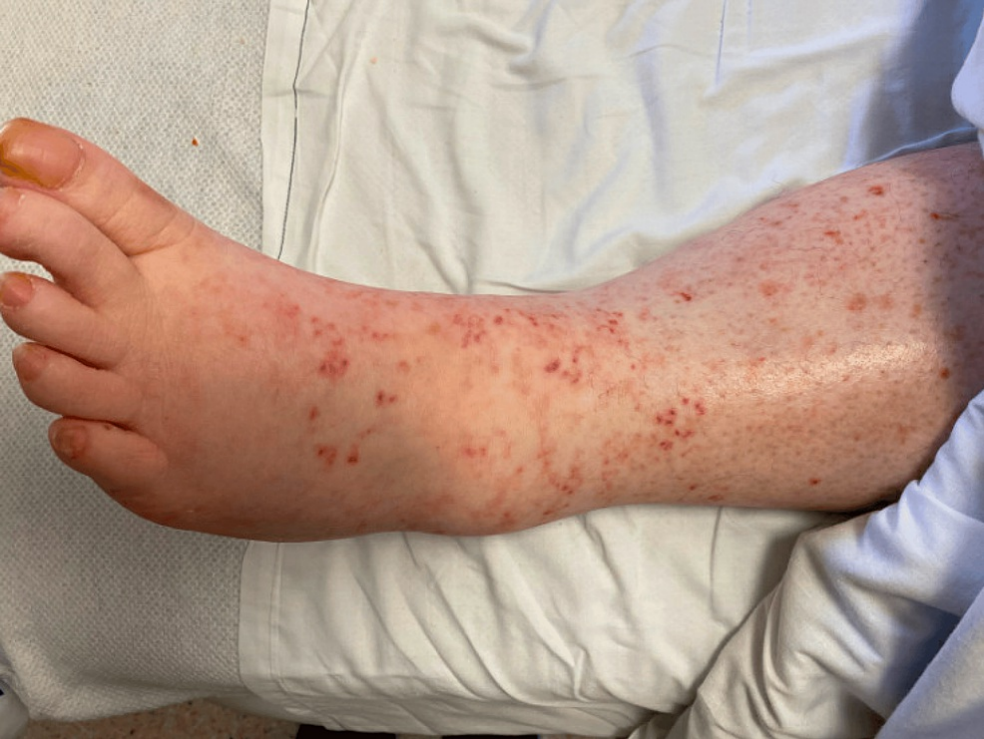
Dermatologic findings reveal skin papules upon patient presentation.

This case centers around a 51-year-old female with a medical history encompassing diabetes and hypertension. Having been on metformin for two years and hydralazine (40mg twice a day) for 13 months, the patient sought hospital care due to a two-month history of a pruritic rash. The delay in seeking medical attention was linked to concerns about introducing more medications to her regimen. The physical examination revealed a distinct rash characterized by arcuate and annular palpable purpura in a reticulate pattern, predominantly on the upper and lower extremities, with a smaller area below the umbilicus. Notably, there was no involvement of the palms, soles, or face (Figure 1).

The patient denied experiencing fever, rashes, weight loss, joint pain, or difficulty swallowing or breathing. She also did not recall any recent sickness, and her infectious workup returned negative. Laboratory results indicated pancytopenia and positive findings for various antibodies (anti-nuclear antibody (ANA), double-stranded DNA, anti-histone antibody, perinuclear anti-neutrophil cytoplasmic antibodies (p-ANCA), cytoplasmic ANCA (c-ANCA), rheumatoid factor), alongside elevated C-reactive protein (CRP) and decreased C3 and C4 levels (Table 1).

**Table 1:** Key laboratory values upon patient presentation K: kilo; M: million; mg: microgram; g: gram; mL: milliliter; uL: microliter; dL: deciliter; L: liter; U: unit; IU: international unit; AI: antibody index

Lab Name	Lab Value	Reference Range
White Blood Cell (WBC)	1.9 K/uL	4-11 K/uL
Red Blood Cell (RBC)	3.1 M/uL	3.7-5.4 M/uL
Hemoglobin	7.8 g/dL	12-16 g/dL
Platelets	113 K/uL	130-450 K/uL
C-Reactive Protein	72 mg/L	<4.9 mg/L
C3, Complement	72 mg/dL	90-180 mg/dL
C4, Complement	8 mg/dL	16-47 mg/dL
Total Complement (CH50)	29 U/mL	31-60 U/mL
Anti-Nuclear Antibody	Positive 1:1280	<1:40
Anti-Histone Antibody	Positive 3.3 U	<1 U
dsDNA Antibody	Positive 16.0 IU/mL	<4 IU/mL
Rheumatoid Factor	Positive 85 IU/mL	<13 IU/mL
Immunoglobulin G	1017 mg/dL	694-1618 mg/dL
Immunoglobulin A	129 mg/dL	81-463 mg/dL
Immunoglobulin M	629 mg/dL	48-271 mg/dL
Antineutrophil Cytoplasmic Antibody (ANCA)	Positive 1:640	<1:20
Myeloperoxidase Antibody (p-ANCA)	Positive 1.6 AI	<0.9 AI
Proteinase-3 Antibody (c-ANCA)	Positive 790.8 AI	<1.0 AI
Smooth Muscle Antibodies	Negative	Negative
Ribonucleoprotein Antibody	Negative	Negative
Chromatin Antibody	Negative	Negative
Cyclic Citrullinated Peptide Antibody	Negative	Negative

The patient’s estimated glomerular filtration rate (eGFR), creatinine, and urine studies were unremarkable.

**Figure 2:**
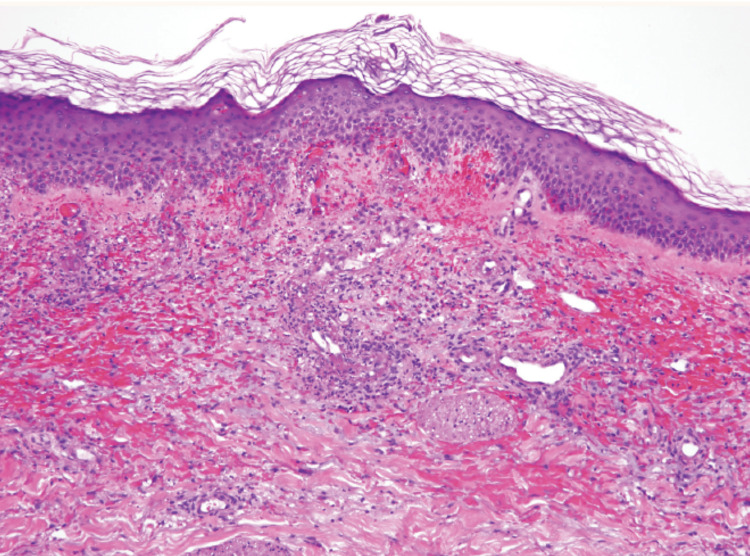
Typical histology from a skin biopsy of leukocytoclastic vasculitis Image adapted from reference #5 with permission

A collaborative approach involving rheumatology, hematology, and dermatology was employed to investigate the unknown etiology of the rash and pancytopenia. Initially suspected of being autoimmune, the lesions prompted considerations of systemic lupus erythematosus, granulomatosis with polyangiitis, or microscopic polyangiitis. Hematology and oncology were consulted to exclude malignancy, yet a bone marrow biopsy revealed no evidence thereof. A 0.6 x 0.5 x 0.4cm punch biopsy conducted by dermatology revealed leukocytoclastic vasculitis (Figure 2).

**Figure 3:**
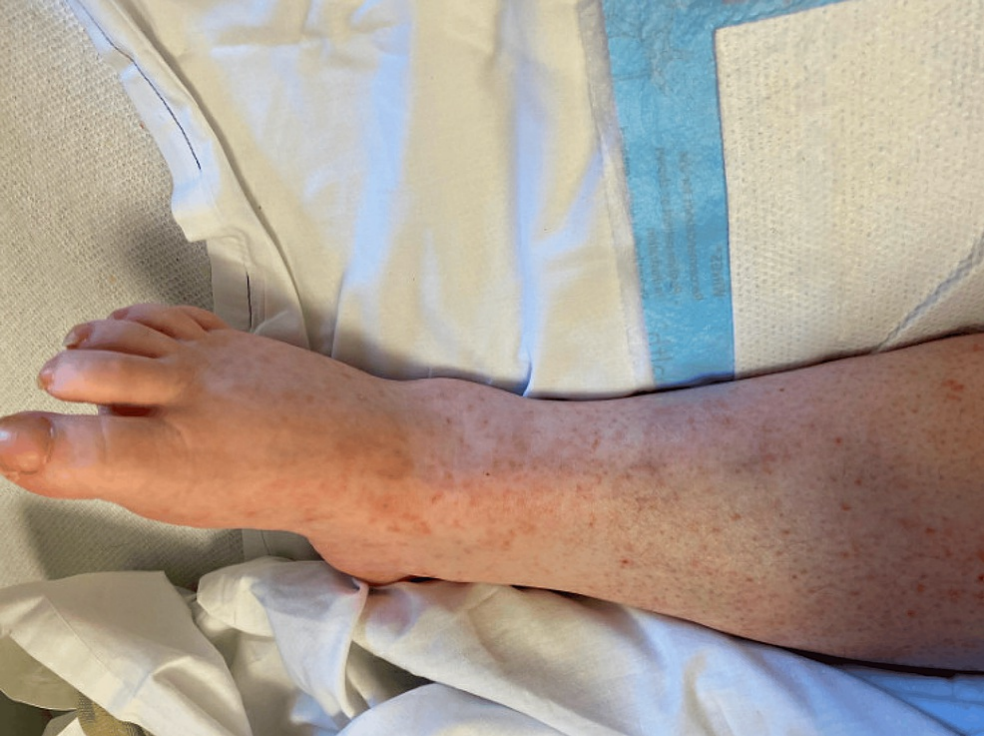
Improvement of dermatologic symptoms with treatment

The rheumatology team attributed this condition to the addition of hydralazine to the patient’s antihypertensive regimen. Promptly, hydralazine was discontinued, and the patient commenced a treatment plan involving glucocorticoids at one gram per kilogram for five days. The tapering of steroids involved a progressive reduction in prednisone dosage over a specific duration, starting with 30mg for seven days, followed by 25mg for 14 days, another 14 days at 25mg, 20mg for 14 days, 15mg for 14 days, 12.5mg for 14 days, 7.5mg for 14 days, and finally, 5mg maintained for two months. The patient was also started on 500mg of mycophenolate mofetil twice a day to address pancytopenia. Plans for outpatient rituximab therapy were formulated, and follow-up appointments demonstrated improvement in the patient’s symptoms with no relapse (Figure 3).

## Discussion

Awareness that hydralazine can induce vasculitis is crucial for healthcare providers due to its potentially severe and life-threatening nature. Vasculitis, characterized by blood vessel inflammation, can lead to complications across various organ systems. Early recognition of hydralazine-induced vasculitis is essential for prompt diagnosis and discontinuation of the medication. Existing studies highlight the varied presentations of hydralazine-associated vasculitis, with ambiguity in its manifestation. This case study presents a unique scenario where the patient exhibited pancytopenia and dermatologic findings without renal or pulmonary involvement, deviating from previous reports [3, 4]. A review of 323 cases revealed 12 patients exposed to hydralazine, all positive for ANAs and ANCA, with variable proteinuria [5]. Notably, this case study, with a shorter duration of hydralazine therapy (13 months versus 22 months) and a younger patient (51 years old versus 70 years old), demonstrated favorable outcomes, emphasizing the impact of early detection on morbidity and mortality. [5] Despite similar antibody and complement findings, the absence of severe complications in this case underscores the importance of timely recognition in improving patient outcomes.

The mechanism by which hydralazine induces ANCA-associated vasculitis remains a subject of ongoing research and has yet to be entirely elucidated. One possibility is that myeloperoxidase (MPO) or proteinase 3 (PR3), which targets neutrophils and monocytes, leads to inflammation of small to medium-sized blood vessels. Another proposed mechanism suggests that hydralazine may act as a hapten, causing a conformational change in neutrophil proteins [2, 6]. This alteration may trigger an immune response, leading to the production of ANCA against neutrophil antigens. The immune system’s recognition of these altered proteins as foreign may contribute to the inflammatory process seen in ANCA-associated vasculitis. Additionally, hydralazine has been associated with drug-induced autoimmunity, where the drug itself or its metabolites may directly stimulate the immune system [2, 6]. This immune activation can lead to the production of ANCA and subsequent vasculitis. Furthermore, genetic predispositions and individual variations in immune responses may play a role in determining why only a small percentage of individuals exposed to hydralazine develop ANCA-associated vasculitis.

This study carries significant importance within the medical field as it illuminates a rare yet potentially severe side effect associated with the widely prescribed medication hydralazine. The identification of hydralazine-induced vasculitis in the presented case underscores the necessity for heightened awareness among healthcare professionals concerning this specific adverse reaction. This newfound knowledge serves to enhance patient safety by facilitating timely intervention, prompt discontinuation of the medication, and the initiation of appropriate treatment strategies. Such actions are instrumental in preventing the further progression of vasculitis and minimizing potential complications. Through the presentation of a detailed case study, this research underscores the critical importance of considering medication-induced complications, especially in patients exhibiting atypical symptoms and those on antihypertensive regimens. The practical implications of this knowledge extend to clinical practice, guiding healthcare providers for the early recognition and proper management of hydralazine-induced vasculitis. Ultimately, this contributes to improved patient outcomes and safety in the realm of hypertension management.

## Conclusions

This study underscores the significance of recognizing and understanding the rare side effects of hydralazine-induced vasculitis. The practical implications of this research extend to healthcare providers, urging heightened awareness of this specific adverse reaction to hydralazine. By disseminating knowledge on the clinical manifestations, diagnostic strategies, and management approaches for hydralazine-induced vasculitis, this study contributes to enhancing hypertension management practices and ultimately leads to improved patient outcomes. Further research in the field of hydralazine-induced vasculitis is warranted to deepen our understanding of the underlying mechanisms and risk factors associated with this rare side effect.
